# Supplementary material for: Temporal Change in Iron Content of Vegetables and Legumes in Australia: A Scoping Review
Source: Foods. 2021 Dec 27;11(1):56. doi: 10.3390/foods11010056 (PMC8750575; doi:10.3390/foods11010056)
Supplement: Supplementary file 1 [file foods-11-00056-s001.zip › foods-1477168-supplementary.pdf]

Supplementary files

Table S1. Contribution of iron intake in Australians by vegetables groups and legumes based on the Australian Health Survey 2011-2 (ABS, 2014)

Vegetable group classified by FSANZ	Varieties included in this review within the vegetable group	% of total iron intake
Potatoes	Coliban potato, Desiree potato, new potato, pale-skinned potato, Pontiac potato, red-skinned potato, Sebago potato	2.6
Other fruiting vegetables	Avocado, bitter melon, capsicum, choko, cucumber, eggplant, hairy melon, mushroom, okra, pumpkin, squash, sweet corn, zucchini	0.9
Leaf and stem vegetables	Artichoke, asparagus, celery, endive, lettuce, rocket, silverbeet, spinach, watercress	0.8
Peas and beans	Bean sprout, green/French bean, green pea, snow pea	0.7
Cabbage, cauliflower and similar brassica vegetables	Bok choy, broccoli, broccolini, Brussels sprouts, cabbage, cauliflower, kale, kohlrabi	0.6
Mature legumes and pulses	Adzuki bean, chick pea, cow pea, dried pea, haricot bean, fava bean, lentil, lima bean, mung bean, navy bean, pigeon pea, red kidney bean, soya bean, sweet lupin, white lupin.	0.4

Table S2. Search strategy used on MEDLINE via Ovid interface for literature on iron content of vegetables

1. exp Australia/
2. Oceania/
3. Australasia/
4. Austral*.tw.
5. Oceani*.tw.
6. "New South Wales".tw.
7. Queensland.tw.
8. "Northern Territory".tw.
9. Victoria*.tw.
10. Tasmania*.tw.
11. NSW.tw.
12. QLD.tw.
13. SA.tw.

14. WA.tw.
15. TAS.tw.
16. NT.tw.
17. ACT.tw.
18. VIC.tw.
19. Sydney.tw.
20. Perth.tw.
21. Adelaide.tw.
22. Melbourne.tw.
23. Brisbane.tw.
24. Darwin.tw.
25. Hobart.tw.
26. Canberra.tw.
27. or/1-26
28. Iron, Dietary/
29. Iron/
30. Nutrients/
31. Trace elements/
32. Micronutrients/
33. Nutritive value/
34. iron.tw.
35. Fe.tw.
36. ((mineral* or nutri* or food*) adj2 (content or composition or level* or concentration* or value*)).tw.
37. micronutrient*.tw.
38. "trace element*".tw.
39. ((physicochemical or chemical or nutri*) adj2 (propert* or parameter*)).tw.
40. "diet* mineral*".tw.
41. or/28-40
42. Vegetables/
43. Plants, Edible/
44. Crops, Agricultural/
45. Solanum tuberosum/
46. Capsicum/
47. Abelmoschus/
48. Zea mays/
49. Cucumis sativus/
50. Cucurbita/
51. Agaricales/
52. Agaricus/
53. Persea/
54. Solanum melongena/
55. Momordica charantia/
56. Spinacia oleracea/
57. Beta vulgaris/
58. Asparagus Plant/
59. Lettuce/

60. Cynara scolymus/
61. Brassica/
62. Medicago sativa/
63. vegetable*.tw.
64. plant-based.tw.
65. potato*.tw.
66. "Solanum tuberosum".tw.
67. capsicum*.tw.
68. pepper*.tw.
69. okra.tw.
70. "lad* finger*".tw.
71. "Abelmoschus esculentus".tw.
72. (sweet adj corn*).tw.
73. sweet corn*.tw.
74. "Zea mays L".tw.
75. cucumber*.tw.
76. "Cucumis sativus".tw.
77. zucchini.tw.
78. "Cucurbita pepo".tw.
79. pumpkin*.tw.
80. squash*.tw.
81. mushroom*.tw.
82. "Agaricus bisporus".tw.
83. avocado*.tw.
84. "Persea americana".tw.
85. chil?i*.tw.
86. choko*.tw.
87. chayote*.tw.
88. "Sechium edule".tw.
89. eggplant*.tw.
90. aubergine*.tw.
91. "Solanum melongena".tw.
92. "bitter melon*".tw.
93. "balsam pear*".tw.
94. gourd*.tw.
95. "Momordica charantia".tw.
96. "hairy melon*".tw.
97. "fuzzy melon*".tw.
98. "Benincasa hispida".tw.
99. spinach.tw.
100. "Spinacia oleracea".tw.
101. silverbeet.tw.
102. chard.tw.
103. "Beta vulgaris cicla".tw.
104. watercress.tw.
105. "Nasturtium officinale".tw.
106. endive*.tw.

107. "Cichorium endivia".tw.
108. asparagus.tw.
109. rocket.tw.
110. "Eruca sativa".tw.
111. lettuce.tw.
112. "Lactuca sativa".tw.
113. celery.tw.
114. "Apium graveolens".tw.
115. "bamboo shoot*".tw.
116. "Phyllostachys edulis".tw.
117. artichoke*.tw.
118. "Cynara scolymus".tw.
119. broccoli*.tw.
120. cauliflower.tw.
121. cabbage*.tw.
122. kale.tw.
123. "choy sum".tw.
124. "bok choy".tw.
125. kohlrabi.tw.
126. Brassica.tw.
127. "bean sprout*".tw.
128. "Vigna radiata".tw.
129. Alfalfa sprouts.tw.
130. "Medicago sativa".tw.
131. or/42-130
132. 27 and 41 and 131
133. limit 132 to English language

Table S3: Search strategy used on MEDLINE via Ovid interface for literature on iron content of legumes

1. Iron, Dietary/
2. iron/
3. nutrients/
4. micronutrients/
5. trace elements/
6. Nutritive Value/
7. iron.tw.
8. Fe.tw.
9. ((mineral* or nutri* or food*) adj2 (content or composition or concentration* or level* or value*)).tw.
10. micronutrient*.tw.
11. "trace element*".tw.
12. ((physicochemical or chemical or nutri*) adj2 (propert* or parameter*)).tw.
13. "diet* mineral*".tw.
14. or/1-13
15. Fabaceae/

16. exp Plants, Edible/
17. Crops, Agricultural/
18. Peas/
19. Seeds/
20. Soybeans/
21. Vigna/
22. Phaseolus/
23. Cicer/
24. Vicia faba/
25. Lupinus/
26. Lens Plant/
27. legum*.tw.
28. (pea or peas).tw.
29. papilionaceae.tw.
30. (bean or beans).tw.
31. (pod or pods).tw.
32. (seed or seeds).tw.
33. pulses.tw.
34. plant-based.tw.
35. (meat adj2 (analogue or alternative)).tw.
36. soy*.tw.
37. soja.tw.
38. "glycine max".tw.
39. adzuki.tw.
40. mung*.tw.
41. moong.tw.
42. "vigna angularis".tw.
43. haricot.tw.
44. "phaseolus vulgaris".tw.
45. (mungbean or mungbeans).tw.
46. ((green or black or golden or bengal) adj1 (gram or grams)).tw.
47. "vigna radiata".tw.
48. "vigna mungo".tw.
49. (chickpea or chickpeas).tw.
50. garbanzo.tw.
51. "desi chana".tw.
52. "cicer arietinum".tw.
53. "vigna unguiculata".tw.
54. "pisum sativum".tw.
55. faba.tw.
56. fava.tw.
57. lupin*.tw.
58. (lentil or lentils).tw.
59. "lens culinaris".tw.
60. or/15-59
61. exp Australia/
62. Australasia/

63. Oceania/
64. austral*.tw.
65. oceani*.tw.
66. "new south wales".tw.
67. queensland.tw.
68. victoria*.tw.
69. tasmania*.tw.
70. "northern territory".tw.
71. NSW.tw.
72. ACT.tw.
73. QLD.tw.
74. VIC.tw.
75. TAS.tw.
76. WA.tw.
77. NT.tw.
78. SA.tw.
79. Sydney.tw.
80. Canberra.tw.
81. Melbourne.tw.
82. Brisbane.tw.
83. Hobart.tw.
84. Perth.tw.
85. Darwin.tw.
86. Adelaide.tw.
87. or/61-86
88. 14 and 60 and 87
89. limit 88 to English language
